# Clonal integration benefits *Calystegia soldanella* in heterogeneous habitats

**DOI:** 10.1093/aobpla/plae028

**Published:** 2024-05-20

**Authors:** Mingyan Li, Siyu Jiang, Tong Wang, Hui Wang, Lijun Xing, Haimei Li, Yingkun Sun, Xiao Guo

**Affiliations:** College of Landscape Architecture and Forestry, Qingdao Agricultural University, No. 700 Changcheng Road, Qingdao 266109, P.R. China; College of Landscape Architecture and Forestry, Qingdao Agricultural University, No. 700 Changcheng Road, Qingdao 266109, P.R. China; College of Landscape Architecture and Forestry, Qingdao Agricultural University, No. 700 Changcheng Road, Qingdao 266109, P.R. China; College of Landscape Architecture and Forestry, Qingdao Agricultural University, No. 700 Changcheng Road, Qingdao 266109, P.R. China; College of Landscape Architecture and Forestry, Qingdao Agricultural University, No. 700 Changcheng Road, Qingdao 266109, P.R. China; College of Landscape Architecture and Forestry, Qingdao Agricultural University, No. 700 Changcheng Road, Qingdao 266109, P.R. China; College of Landscape Architecture and Forestry, Qingdao Agricultural University, No. 700 Changcheng Road, Qingdao 266109, P.R. China; College of Landscape Architecture and Forestry, Qingdao Agricultural University, No. 700 Changcheng Road, Qingdao 266109, P.R. China

**Keywords:** *Calystegia soldanella*, clonal division of labour, coast, environmental heterogeneous, soil nutrients

## Abstract

Abstract. Land-use change and tourism development have seriously threatened the ecosystems of coastal protection forests and beaches. Light and nutrients are spatially heterogeneously distributed between the two ecosystems. Clonal plants, such as *Calystegia soldanella*, which play a crucial role in maintaining the ecological stability of coastal habitats, are likely to encounter diverse environments. In this study, we investigated clonal integration and the division of labour in *C. soldanella* under heterogeneous (high nutrient and low light [HNLL]; low nutrient and high light [LNHL]) and homogeneous habitats. We cultivated pairs of connected and severed ramets of *C. soldanella* in these environments. Our results showed the total biomass (TB) of connected ramets was higher than that of severed ramets in heterogeneous environments, suggesting clonal integration enhances growth in heterogeneous habitats. The root shoot ratio was significantly lower in HNLL than in LNHL conditions for connected ramets, demonstrating a division of labour in growth under heterogeneous conditions. However, parameters of clonal propagation of *C. soldanella* did not significantly differ between connected and severed ramets in heterogeneous environments, indicating no division of labour in clonal propagation. In homogeneous environments, the growth of *C. soldanella* did not benefit from clonal integration. Connected ramets in heterogeneous habitats exhibited higher TB than in homogeneous habitats. The TB of one ramet in HNLL was consistently higher than that in LNHL, irrespective of ramet’s states, which suggests that high soil nutrients may enhance the growth. We conclude that *C. soldanella* has the capability of clonal integration to achieve high biomass in heterogeneous but not in homogeneous conditions, and the establishment of coastal protection forests (high nutrient and low light) may foster the growth of *C. soldanella*.

## Teaser

*Calystegia soldanella* , as clonal plants, which play a crucial role in maintaining the ecological stability of coast habitats, are likely to encounter diverse environments. In this study, we investigated clonal integration and the division of labour in *C. soldanella* under heterogeneous (high nutrient and low light; low nutrient and high light) and homogeneous habitats. We found that *C. soldanella* has the capability of clonal integration to achieve high biomass in heterogeneous but not in homogeneous conditions, and the establishment of coastal protection forests (high nutrient and low light) may foster the growth of *C. soldanella* .

## Introduction

Environmental resources such as soil nutrients and light are crucial abiotic factors for plant survival (Uddin and Robinson 2018; Shtein *et al.* 2021; Wang *et al.* 2021a). In natural habitats, the distribution of soil resources and light often varies, influencing the growth and reproduction of plants at relevant scales (Kumar *et al.* 2018; Shen *et al.* 2019; Adomako *et al.* 2021). Conversely, some natural environments exhibit homogeneity, which also impacts plant growth (Zhang *et al.*, 2016; Dong *et al.* 2022). Clonal plants consist of numerous potentially independent units, known as ramets, interconnected by stolons or rhizomes (Huang *et al.* 2018; Li *et al.* 2018; Shtein *et al.* 2021). Given their robust growth and reproduction capabilities, clonal plants are likely to colonize extensive areas, frequently encountering both heterogeneous and homogeneous environments (Hutchings *et al.* 2003; Huang *et al.* 2018). Therefore, investigating how clonal plants adapt to these complex environmental distributions is essential for understanding their growth dynamics under varying resource conditions.

Environmental resource heterogeneity is a key driver for the growth of clonal plants (Wang *et al.* 2021a). Clonal integration, the process by which is the sharing of resources by connected modules of a clonal system share resources, occurs in heterogeneous habitats (Roiloa *et al.* 2014; Ma *et al.* 2023). Typically, resources are transferred from ramets in resource-rich areas to those in resource-poor areas, enhancing the growth and survival of the latter (Luo *et al.* 2015; Shi *et al.* 2022). In other words, clonal plants leverage heterogeneous resources through clonal integration, increasing their resilience to resource variability (Wang *et al.* 2017; Lu *et al.* 2020). When the availability of two critical resources is inversely correlated spatially, clonal integration can facilitate a division of labour, enhancing the efficiency of resource use in clonal plants (Wang *et al.* 2011; Roiloa *et al.* 2016; Huang *et al.* 2018; Shtein *et al.* 2021). This division of labour is manifested not only in the differential allocation of aboveground and underground biomass but also in the reproductive traits of clonal plants (Zhang *et al.* 2016). Due to the economic benefit of absorbing resources more efficiently in areas where they are abundant, division of labour allows each ramet to specialize in exploiting locally abundant resource (Zhang and Zhang 2013; Wang *et al.* 2016; Lin *et al.* 2018). Furthermore, Xu *et al.* (2012) found that under heterogeneous conditions, *Alternanthera philoxeroides* and *Phyla canescens* exhibit different divisions of labour at morphological and growth levels, suggesting that different species have different mechanisms of clonal integration and division of labour in the patchy environments. Therefore, both clonal integration and division of labour are influenced by species-specific traits.

In homogeneous habitats, clonal integration and division of labour are also exhibit by some clonal plants (Xi *et al.* 2019; Dong *et al.* 2022). Zhang *et al.* (2016) demonstrated that clonal integration is generally more beneficial than clonal fragmentation in environments with uniformly high nutrients. However, some studies have suggested that clonal integration might be less disadvantageous in homogeneous environments (Caraco and Kelly 1991; Alpert 1999). Hence, the impact of homogeneous resource environments on the clonal integration and division of labour of clonal plants remains a subject of ongoing debate.

In recent years, land-use change and tourism development have seriously damaged the beach environments, posing threats to coastal ecosystems (Yi *et al.* 2018; Defeo *et al.* 2021). *Calystegia soldanella*, a perennial native clonal herb, is widely distributed along coastal beaches in China (Wang *et al.* 2021b). And *C. soldanella* exhibits robust sand-fixation capabilities, playing a crucial role in maintaining the ecological stability of sandy beaches (Ohsako and Matsuoka 2008; Di Sacco and Bedini 2014). Previous studies have primarily focused on the extracts, physiological and morphological characteristics, and population composition of *C. soldanella* (Carmelina *et al.* 2013; Zuccarini *et al.* 2015; Kim *et al.* 2022). Consequently, we have explored the cloning capabilities of *C. soldanella*, which is beneficial for understanding of its growth, asexual reproduction and population expansion. Observations indicate that some ramets of *C. soldanella* thrive in sandy beaches and rock crevices, characterized by high light and low soil nutrients availability. In contrast, other ramets grow in coastal protection forests, where the environment features low light and high nutrient due to canopy shading and litter decomposition. Therefore, our study investigated clonal integration of *C. soldanella* across these varied environmental conditions and in a homogeneous environment. We tested the following hypotheses: (1) Clonal integration and division of labour in growth and propagation enhance the growth performance of *C. soldanella* in heterogeneous habitats when ramets are connected compared to when they are severed. (2) In homogeneous habitats, *C. soldanella* also exhibited the capacity of clonal integration and division of labour. (3) According to previous studies, while clonal plants in heterogeneous environment generally exhibit clonal integration and division of labour, clonal integration may sometimes be disadvantageous in homogeneous environments. Hence, when ramets are connected, the biomass of *C. soldanella* in heterogeneous treatments is expected to be higher than that in homogeneous treatments.

## Materials and Methods

### Species selection and cultivation

The study was conducted in the greenhouse at Qingdao Agricultural University, located in Chengyang District, Qingdao City, Shandong Province, China (36° 31ʹ N, 120° 39ʹ E). This region experiences a temperate monsoon climate, characterized by an annual average temperature of 12.6 °C. The coldest month is January, with an average temperature of −2 °C, while the hottest month is August, averaging 25.7 °C. The mean annual precipitation is approximately 700 ± 100 mm (Chen *et al.* 2024). The experiment was conducted in a greenhouse with a steel pipe frame covered by a plastic film to prevent precipitation. During the experiment, ventilation was maintained by rolling up the sides of the plastic film.

On 1 July 2019, clonal fragments of *C. soldanella*, each with similar size and rhizome length, were collected from Yangkou Beach (36° 25ʹ N, 120° 68ʹ E), located in Laoshan District, Qingdao City, Shandong Province. These fragments, consisting of two contiguous ramets, were sourced equally from beach areas (high light and low nutrient environment) and coastal protection forests (low light and high nutrient environment). After collection, the clonal fragments were grown in the greenhouse for two weeks to acclimate to the greenhouse environment. The clonal fragments were then planted in boxes (without dividers) filled with the same substrate used in the experiments. Subsequently, 40 clonal fragments of uniform size were selected for the study, each ramet featuring four leaves and fibrous roots was about 8cm in length.

## Experimental design

On 15 July 2019, we planted each ramet of clonal fragments into two equal parts (16 cm long × 18 cm wide × 8 cm deep) of rectangular plastic boxes (32 cm long × 18 cm wide × 8 cm deep) with a plastic divider in the middle. A 0.5 cm × 0.5 cm opening was cut in the centre of the plastic divider to allow the rhizomes to pass through. After positioning the ramets, with the connecting rhizome going through the opening in the divider, the opening was sealed with sea mud to inhibit the transfer of water and nutrients between the compartments. Subsequently, each plastic box was filled with 5 kg of the substrate, a 3:1 (v/v) mixture of cleaned river sand and sterilized soil.

We established three experimental nutrient levels: (1) low-nutrient (LN), where no slow-release fertilizer was added; (2) medium-nutrient (MN), where 0.2 g of slow-release fertilizer (N:P:K = 20:20:20, The Scotts Company, LLC., Marysville, OH, USA) was added weekly; and (3) high-nutrient (HN), where 0.4 g of slow-release fertilizer was added weekly. The LN treatment mimicked the nutrient conditions typical of beaches, while the HN treatment replicated those of coastal protection forests.

We also established three experimental levels of light availability: (1) low-light (LL), where the area was covered with two layers of woven black nylon nets, reducing the light intensity to 10–15% of the natural full light; (2) medium-light (ML), where the area was covered with a single layer of woven black nylon net, reducing the light intensity to 45–50% of the natural full light; and (3) high-light (HL), where the area was exposed to the full light available in the greenhouse, approximately 85–90% of the natural full light. The LL treatment (10–15%) simulated the light conditions of a coastal protection forest, while the HL treatment (85–90%) replicated the light conditions of an unsheltered beach. To ensure consistency in the total amount of light between the heterogeneous and homogeneous environment, the medium-light treatment was adjusted to the average light intensity of the HL and LL treatments. Light intensity for all treatments was calibrated using a Quantum/Foot-Candle Meter (Spectrum Technologies, Inc., USA).

**Figure 1.**
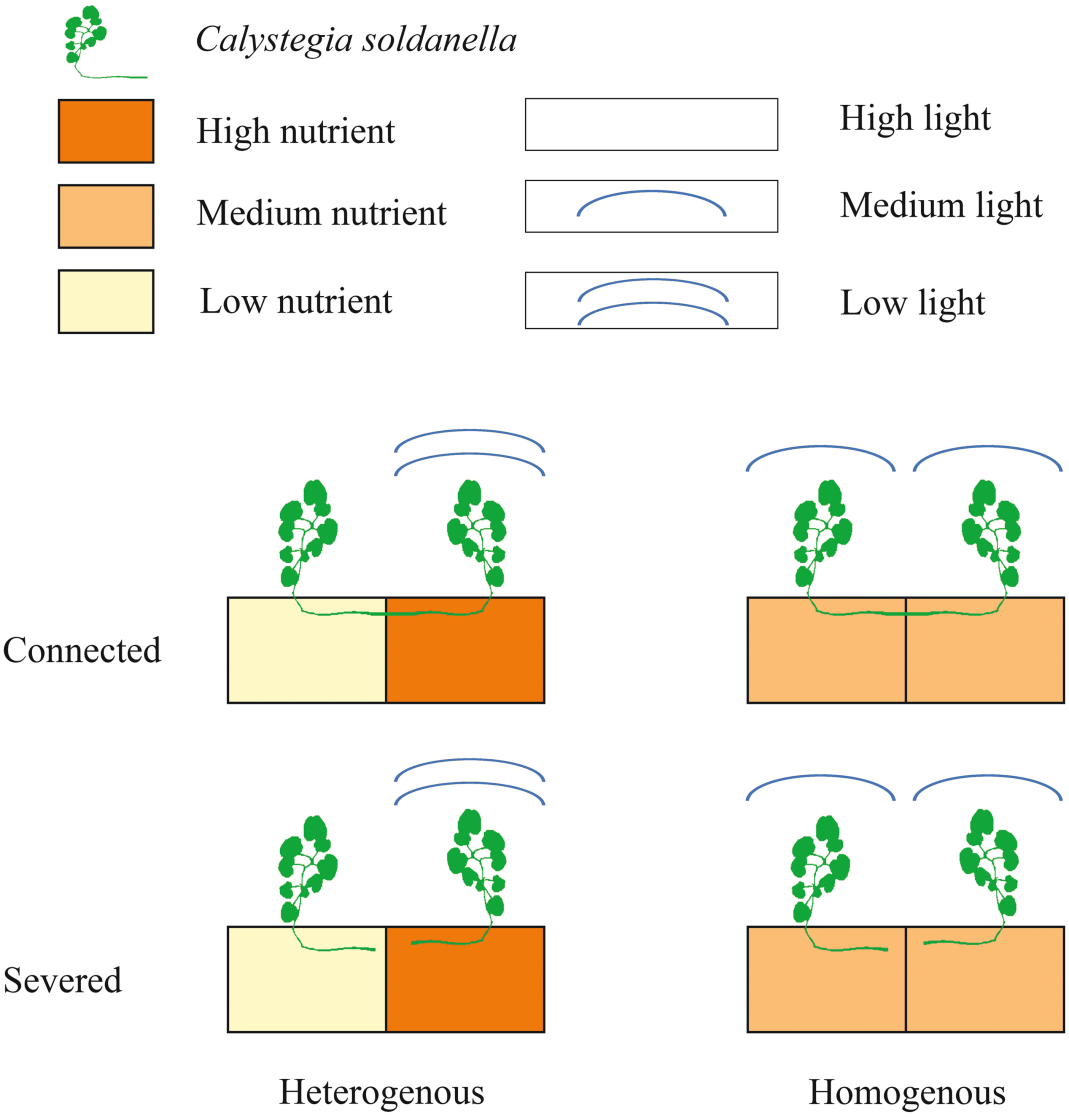
Schematic representation of the experimental design. Clonal fragments consisting of two ramets were grown under either heterogeneous or homogeneous conditions. In heterogeneous conditions, one ramet had high soil nutrient availability and low light (two circular arcs); the other ramet had low soil nutrient availability and high light (no circular arc). In homogeneous conditions, the soil nutrient availability was medium and the light was medium (one circular arc). The two clonal fragments were grown with or without the rhizome connections.

The clonal fragments were randomly assigned to either a reciprocal heterogeneous or a homogeneous treatment (Fig. 1). In the heterogeneous HNLL-LNHL, one ramet from each clonal fragment was placed in the HNLL treatment, and the other in the LNHL treatment. For the homogeneous treatments, both interconnected ramets of a single clonal fragment were uniformly subjected to the MNML-MNML treatment. In both heterogeneous and homogeneous environments, ramets states had two types of treatments: the two ramets were either connected or severed. Throughout the experimental period, the total light and nutrient supply remained constant.

Each treatment consisted of 10 replicate boxes (20 pots), totalling 40 boxes (80 pots). During the experiment, soil moisture content was daily monitored using a soil moisture meter (DL-TH20, Hangzhou Gsome Technology Co., China), and watering was adjusted to maintain a soil water content of approximately 25%, similar to that at the collection site of the plants. Forty boxes were organized into four treatments, with each treatment consisting of 10 boxes. These boxes were allocated across 10 blocks, with each box in a treatment assigned to a different block. This configuration helps mitigate the impact of confounding factors. The experiment was conducted over 49 days, from 15 July 2019, to 1 September 2019.

## Measurements

### Clonal propagation parameters

Prior to harvest, the number of newly generated ramets (aboveground ramets) and the length of each ramet were recorded in the two parts of each pot (16 pots in each treatment). And the average aboveground ramet length was then calculated for each pot and each box. At harvest, two parts of each pot (16 pots in each treatment) were harvested separately. The root was cleaned with tap water. ‘Spacers’, which are rhizomes produced by each ramet and ‘underground ramets’, which have developed but not emerged above ground. We recorded the spacers number, spacers length and underground ramets number. The average spacer length (spacer length) were calculated for each pot and each box.

### Clonal growth parameters

Post-harvest, the plants were divided into rhizomes, roots, stems, and leaves. The plant material from the 16 pots in each treatment was initially placed in a 105 °C oven for 0.5 h and subsequently dried at 85 °C for 48 h. The dry weight of each part was weighed and the following calculations were made:


$$\begin{array}{lll} Totalbiomass= & \;rootbiomass+stembiomass\; & +leafbiomass+rhizomebiomass; \end{array}$$

$$\begin{array}{lll} Rootshootratio\left(RSR\right)= & rootbiomass/\; & (leafbiomass+stembiomass). \end{array}$$

## Data analysis

Linear mixed models were applied to test the effects of environment, ramets states and their interaction on each parameter of the *C. soldanella*. For whole clonal plant parameters, we included environment (heterogeneous vs homogeneous), ramets states (connected vs severed) and their interaction as fixed effects, with block serving as a random effect. For clonal ramets parameters, the same fixed effects were applied, with the whole clonal plant considered a random effect. Turkey’s multiple range tests with a Bonferroni correction were conducted to identify significant differences among treatments within each box (the whole clonal plant) and to correct for multiple comparisons and control Type I error across different treatments. Paired *T*-tests were used to compare the parameters of connected or severed ramets within both heterogeneous and homogeneous environments. Before applying ANOVAs, the data were tested for normality and homogeneity of variance. if necessary, the data were log-transformed to increase normality and homogeneity of variances. All statistical analyses were performed using IBM SPSS Statistics 25.0 (IBM Corp., Armonk, NY, USA). Figures were drawn using Origin 2019 software (Origin Lab Co., MA, USA).

## Results

**Figure 2.**
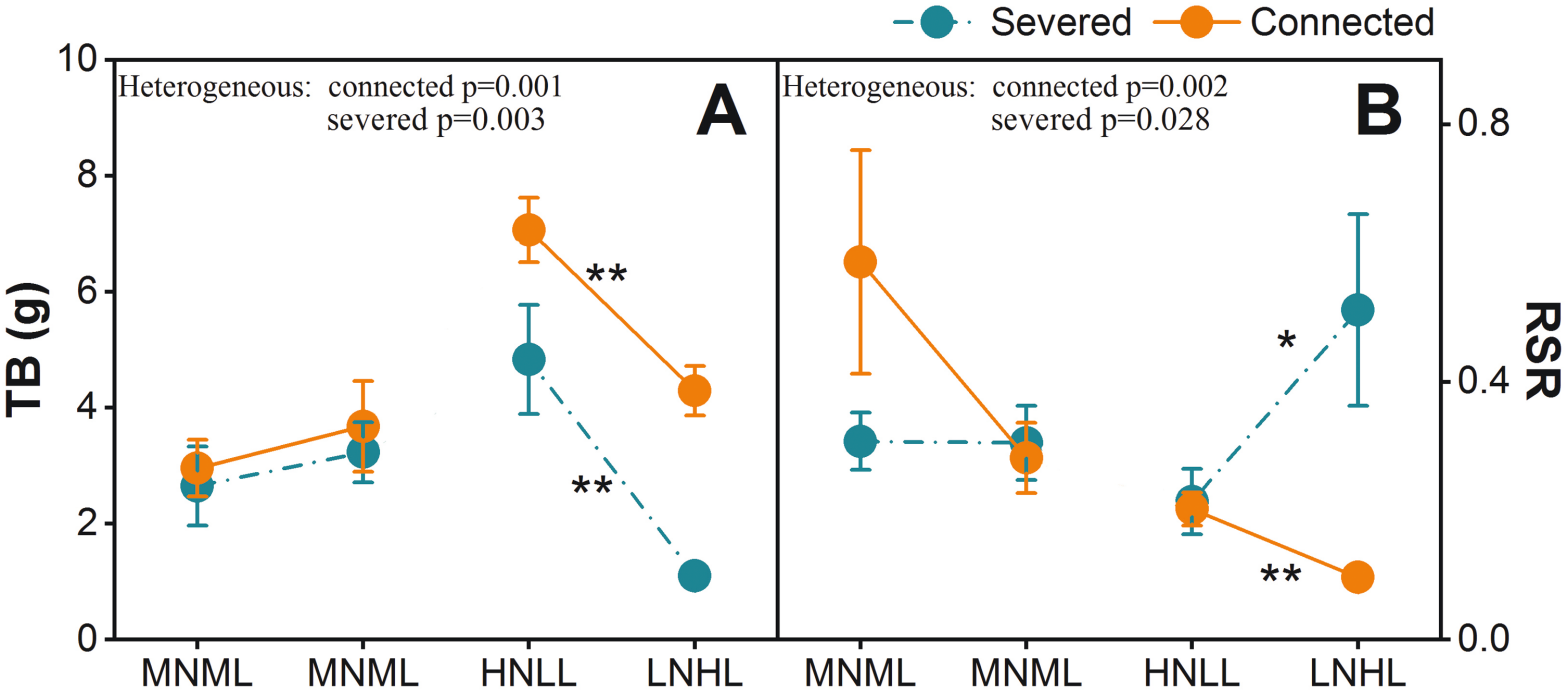
TB (total biomass) and RSR (root shoot ratio) (mean ± SE, *n* = 8) for two parts of the pot under homogeneous and heterogeneous conditions. ** denotes significant differences between the two ramets that connected or severed at *p* ≤ 0.01 by paired T-test. LNHL: low soil nutrient and high light conditions, HNLL: high soil nutrient and low light conditions, MNML: medium soil nutrient and medium light conditions.

In heterogeneous environments, the total biomass (TB) of *C. soldanella* in the connected state was higher than in the severed state (Table 1; Fig. 2A). Regardless of whether the ramets were connected or severed, TB was significantly higher in the high nutrient and low light (HNLL) condition compared to the low nutrient and high light (LNHL) condition (Fig. 2A). When the ramets were severed, the root-to-shoot ratio (RSR) in the HNLL treatment was significantly lower than that in the LNHL treatment (Table 1; Fig. 2B). Conversely, when the ramets connected, RSR in the LNHL treatment was significantly lower than in the HNLL treatment (Fig. 2B). For both connected and severed states, the parameters of clonal propagation of *C. soldanella* showed no significant difference between the HNLL and LNHL treatments (see Supporting Information—Fig. 1).

**Table 1:** Results of generalized linear mixed models for effects of environment, ramets states and their interaction on the TB and RSR of ramets of *Calystegia soldanella*

		TB (g)		RSR	
Effect	*df*	*F*	*P*	*F*	*P*
Fixed factor					
E	1	2.487	0.12	11.725	**0.001**
R	1	5.437	**0.023**	8.874	**0.004**
E × R	1	5.066	**0.028**	7.306	**0.009**
Random factor					
Whole clonal plant	1	0.332	0.332	9.268	**0.003**

E: environment; R: ramets states; TB: total biomass; RSR: root shoot ratio.

Significant results with *P* value < 0.05 are shown in bold.

In homogeneous environments, there were no significant differences in any measured parameters of *C. soldanella* between connected and severed states (Fig 2; see Supporting Information—Fig. 1).

**Figure 3.**
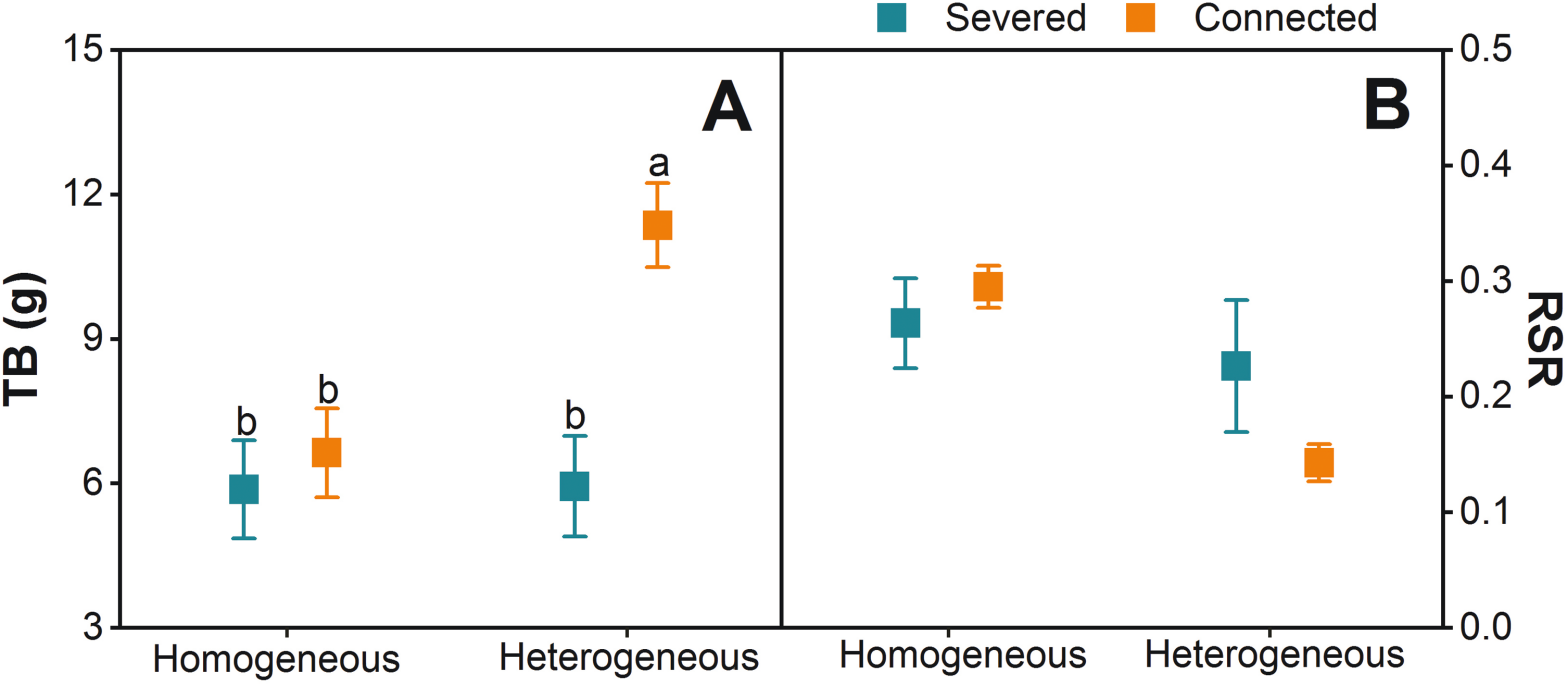
TB (total biomass) and RSR (root shoot ratio) (mean ± SE, *n* = 8) for the whole boxes under homogeneous and heterogeneous conditions. Different letters denote significant differences at correction *P* ≤ 0.05 by Turkey’s test with Bonferroni correction.

When in connected states, TB in heterogeneous environments was significantly higher than in homogeneous environments; however, in severed states, TB were not significantly different between in heterogeneous and homogeneous conditions (Table 2; Fig 3). Irrespective of the ramets being connected or severed, the parameters of clonal propagation and RSR showed no significant differences between heterogeneous and homogeneous environments (Fig 3; see Supporting Information—Fig. 2).

**Table 2:** Results of generalized linear mixed models for effects of environment, ramets states and their interaction on the TB and RSR of whole clone of *Calystegia soldanella*

		TB (g)		RSR	
Effect	*df*	*F*	*P*	*F*	*P*
Fixed factor					
E	1	9.141	**0.005**	10.178	**0.004**
R	1	4.412	**0.045**	0.916	0.347
E × R	1	3.261	0.082	3.473	0.073
Random factor					
Block	1	2.014	0.165	11.48	**0.002**

E: environment; R: ramets states; TB: total biomass; RSR: root shoot ratio.

Significant results with *P* value < 0.05 are shown in bold.

## Discussion

Our results confirmed that *C. soldanella* exhibited higher biomass when the ramets were connected compared to when they were severed in the heterogeneous habitats, supporting our first hypothesis. This aligns with previous studies indicating that clonal plant integration promotes ramet growth in heterogeneous habitats (Dong *et al.* 2015; Shen *et al.* 2019; Shi *et al.* 2021). When the availabilities of two essential resources is negatively correlated, the connected ramets specialize in absorbing the more abundant resources available to each ramet portion, as plants have high efficiency in absorbing resources at high-resource conditions (Roiloa *et al.* 2014). Compared to severed ramets, resource exchange between connected ramets leads to greater overall resource availability and higher photochemical efficiency, thereby facilitating biomass accumulation in clonal plants (Roiloa *et al.* 2013). Therefore, in our study, *C. soldanella* was able to translocate resources among ramets in an environment with reciprocal resource distribution through clonal integration.

Regarding the root-shoot ratio, our findings indicate that *C. soldanella* exhibits a division of labour in growth in heterogeneous habitats, consistent with our first hypothesis. In our study, when the ramets were severed, one ramet displayed a low root shoot ratio in high nutrient and low light treatments, suggesting enhanced aboveground growth due to limited light. The other ramet exhibited a high root shoot ratio in low nutrient and high light treatments, indicating increased underground growth due to lack of nutrients. However, the root shoot ratio in connected ramets in high nutrient and low light conditions was significantly lower than in low nutrient and high light conditions, suggesting resource sharing and division of labour between two connected ramets in heterogeneous conditions. Previous studies have also shown that when ramets were connected, one ramet in high nutrient and low light treatments increases root investment, enhancing soil nutrient resources, while another ramet in low nutrient and high light treatments reduces the root shoot ratio, favouring photosynthesis (Roiloa *et al.* 2014; Estrada *et al.* 2020; Portela *et al.* 2021). This specialization allows each ramet to exploit the most abundant local resource, conferring advantages to the whole clone (Roiloa *et al.* 2016). This phenomenon is consistent with observations in other clonal plants, such as *Mikania micrantha* and *Alternanthera philoxeroides* (Zhang *et al.* 2016; Huang *et al.* 2018).

In our study, there were no significant differences in clonal propagation parameters, such as aboveground ramet number, spacer length, and spacer number, of *C. soldanella* in heterogeneous environments, regardless of whether the ramets were connected or severed. This result suggests that *C. soldanella* did not exhibit a division of labour in clonal propagation in heterogeneous habitats, contradicting our first hypothesis. This finding is also inconsistent with previous studies which demonstrated that connected apical younger ramets typically invested more resources in generating new ramets, thus enhancing clonal propagation and lateral expansion. In contrast, basal older ramets tend to allocate more resources in root development, thereby absorbing more nutrition to support the growth of other ramets (Roiloa *et al.* 2013; Xi *et al.* 2019). One possible explanation for the discrepancy between our results and prior findings is that our study did not differentiate between older and younger ramets of *C. soldanella*.

In homogeneous environments, our experiment revealed no significant differences across all parameters between the two ramets, whether the ramets were connected or not, indicating that *C. soldanella* did not demonstrate clonal integration or clonal division of labour when the cloned fragments were connected in such habitats. This result is in contrary with our second hypothesis. Previous studies has shown that the division of labour is typically subdued in environments with low resources, while clonal plants exhibit significant clonal division of labour in high resources conditions (You *et al.* 2016; Zhang *et al.* 2016). Thus, except for those related to species traits, our findings might be influenced by the levels of light and soil nutrients used in our experiments. Regrettably, we did not establish variable resource levels in homogeneous environments for this experiment. In future studies, we plan to incorporate diverse resource levels in homogeneous settings to further explore the growth mechanism of *C. soldanella*.

When the ramets were connected, the total biomass in heterogeneous habitats was higher than in homogeneous habitats, supporting our third hypothesis. However, other studies have indicated that clonal plants can benefit from clonal integration in both heterogeneous and homogeneous habitats (You *et al.* 2016; Wang *et al.* 2021a). In our study, the primary reason for this result appears to be the clonal integration and division of labour in growth of *C. soldanella* within the heterogeneous environments. Our findings suggest that the heterogeneous habitats of coastal protection forests and beaches are conducive to the population growth and expansion of *C. soldanella*.

Interestingly, regardless of whether the ramets were connected, the total biomass and aboveground ramet length in high nutrient and low light treatments were significantly higher than those in low nutrient and high light treatments. This phenomenon may be linked to the specific light and nutrient settings in our experiment. If future experiments modify these conditions, such as by increasing the light gradient or decreasing the difference in soil nutrient level, opposite results may be observed. Our findings imply that coastal protection forests significantly enhance the growth of *C. soldanella*, as these environments can provide high nutrition through litter decomposition and reduced light due to canopy shading, compared to beach habitats (Xie *et al.* 2018).

## Conclusion

Clonal integration in the heterogeneous environments resulted in higher biomass of *C. soldanella*. According to the root shoot ratio, we observed a division of labour in clonal growth in heterogeneous treatments when ramets were connected. However, *C. soldanella* had a weak division of labour in clonal propagation, potentially because we did not differentiate between older and younger ramets, which may have reduced our ability to detect divisions of labour in this study. In addition, our results indicated no division of labour of *C. soldanella* in homogeneous habitats, which could be due to species-specific traits and the levels of light and soil nutrients in our experiment. When the ramets were connected, *C. soldanella* exhibited higher total biomass in heterogeneous habitats than in homogeneous habitats. Notably, our study found that growth in high nutrient and low light conditions was greater than in low nutrient and high light conditions, regardless of the ramet states. This result may be attributed to the experimental setting for light and nutrients. Therefore, we conclude that *C. soldanella* is capable of clonal integration in heterogeneous conditions, and suggest that enhancing soil nutrient levels and appropriately reducing light intensity could promote its growth. In future research, we plan to conduct long-term experiments and distinguish between apical and basal ramets to further explore the growth mechanism of *C. soldanella* in various environments.

## Supporting Information

The following additional information is available in the online version of this article –

**Figure S1.** Spacer number, Spacer length, Aboveground ramet number, Aboveground ramet length and Underground ramet number (mean ± SE, n = 8) for two parts of the pot under homogeneous and heterogeneous conditions. ** denotes significant differences at *p* ≤ 0.01 between the two ramets that were connected or severed by paired *T*-test. LNHL: low soil nutrient and high light conditions, HNLL: high soil nutrient and low light conditions, MNML: medium soil nutrient and medium light conditions.

**Figure S2.** Spacer number, Spacer length, Aboveground ramet number, Aboveground ramet length and Underground ramet number (mean ± SE, n = 8) for the whole boxes under homogeneous and heterogeneous conditions. Different letters denote significant differences at correction *p* ≤ 0.05 by Turkey’s test with Bonferroni correction.

**Table S1** Results of generalized linear mixed models for effects of environment, ramet states and their interaction on the growth index of ramets of *Calystegia soldanella.*

**Table S2** Results of generalized linear mixed models for effects of environment, ramet states and their interaction on the growth index of the whole clone of *Calystegia soldanella.*

plae028_suppl_Supplementary_Figure_S1

plae028_suppl_Supplementary_Figure_S2

plae028_suppl_Supplementary_Table_S1

plae028_suppl_Supplementary_Table_S2

## Data Availability

The datasets generated and/or analyzed during the current study are available in the figshare repository, https://doi.org/10.6084/m9.figshare.25792902.v1.
