# Supplementary figures and images for: Clonal integration benefits Calystegia soldanella in heterogeneous habitats
Source: AoB Plants. 2024 May 20;16(3):plae028. doi: 10.1093/aobpla/plae028 (PMC11161862; doi:10.1093/aobpla/plae028)

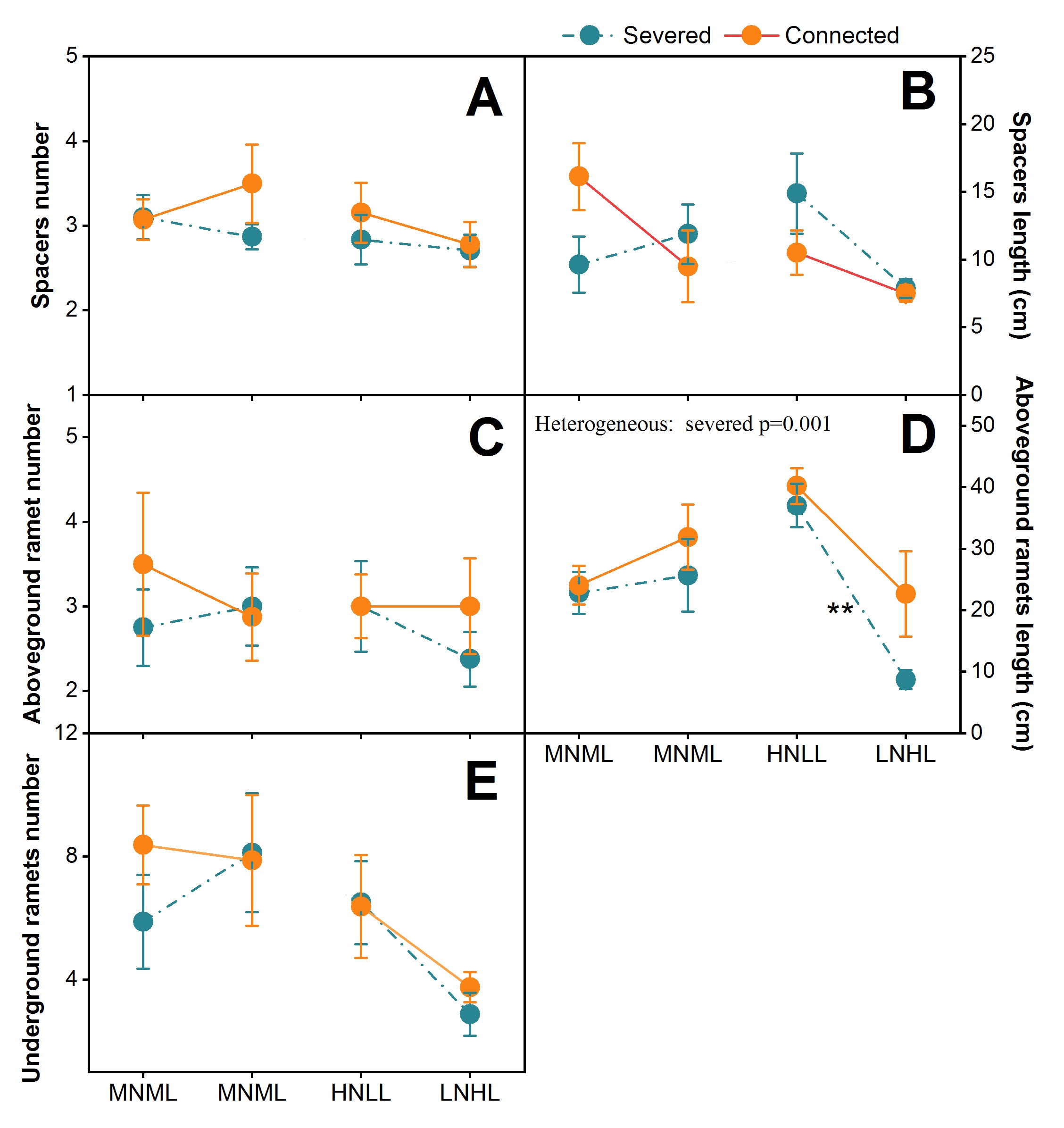

Supplement: plae028_suppl_Supplementary_Figure_S1 [file plae028_suppl_supplementary_figure_s1.jpeg]

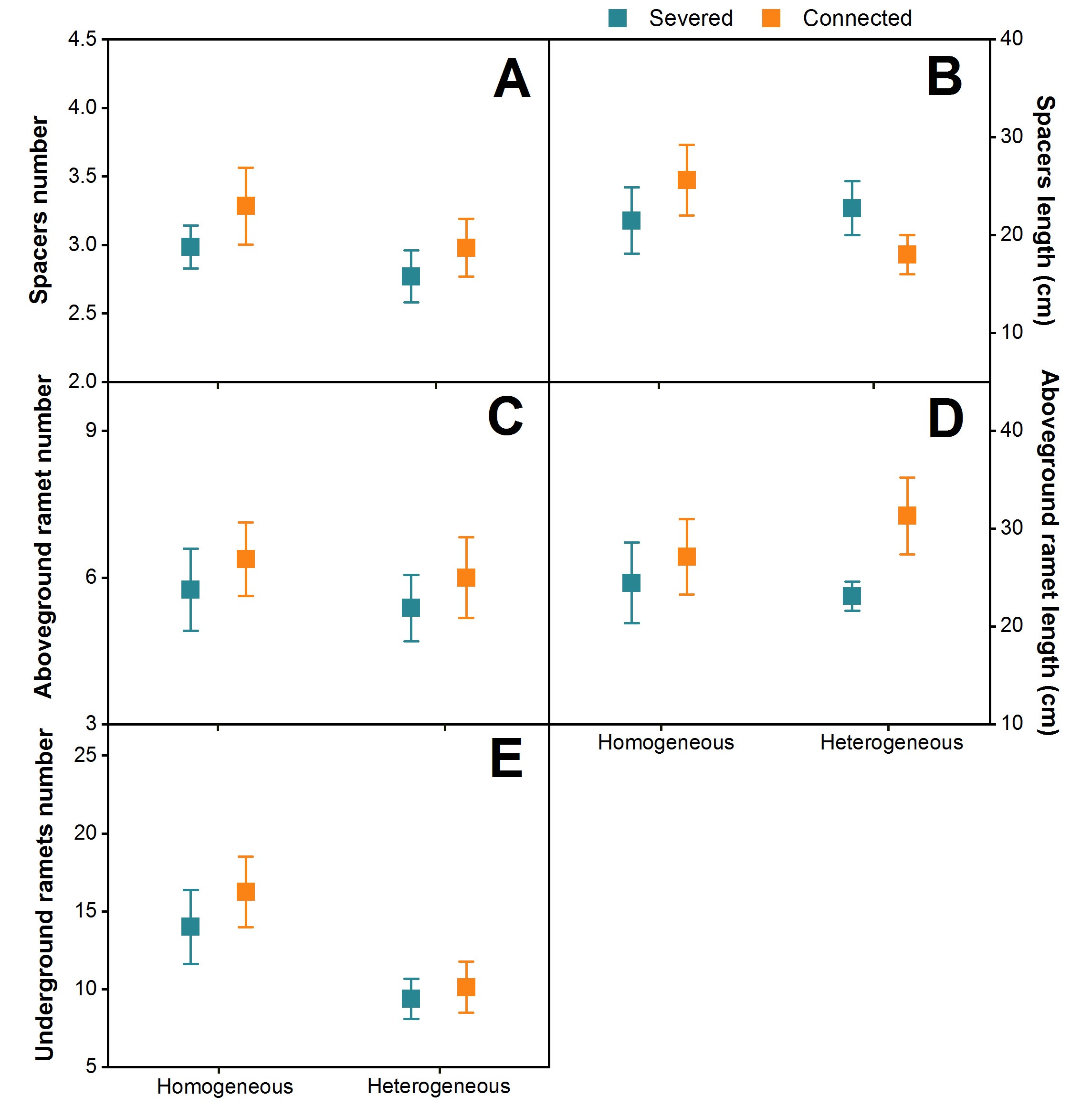

Supplement: plae028_suppl_Supplementary_Figure_S2 [file plae028_suppl_supplementary_figure_s2.jpeg]
