# Supplementary material for: Clonal integration benefits Calystegia soldanella in heterogeneous habitats
Source: AoB Plants. 2024 May 20;16(3):plae028. doi: 10.1093/aobpla/plae028 (PMC11161862; doi:10.1093/aobpla/plae028)
Supplement: plae028_suppl_Supplementary_Table_S1 [file plae028_suppl_supplementary_table_s1.docx]

Table S1 Results of generalized linear mixed models for effects of environment, ramets states and their interaction on the growth index of ramets of *Calystegia soldanella*

|  |  | SN | | SL(cm) | | ARN | | ARL(cm) | | URN | |
| --- | --- | --- | --- | --- | --- | --- | --- | --- | --- | --- | --- |
| Effcet | *df* | *F* | *P* | *F* | *P* | *F* | *P* | *F* | *P* | *F* | *P* |
| Fixed factor |  | | | | | | | | | | |
| E | 1 | 0.039 | 0.844 | 0.273 | 0.604 | 1.595 | 0.212 | 0.776 | 0.382 | 0.011 | 0.916 |
| R | 1 | 0.551 | 0.461 | 0.073 | 0.788 | 2.872 | 0.095 | 0.002 | 0.961 | 1.149 | 0.288 |
| E × R | 1 | 0.065 | 0.8 | 1.969 | 0.166 | 0.000 | 1 | 0.414 | 0.523 | 0.124 | 0.726 |
| Random factor |  | | | | | | | | | | |
| Whole clonal plant | 1 | 0.027 | 0.869 | 0.065 | 0.8 | 2.135 | 0.149 | 0.992 | 0.323 | 0.692 | 0.409 |

E: environment; R: ramets states; SN: spacers number; SL: spacers length; ARN: aboveground ramets number; ARL: aboveground ramets length; URN: underground ramets number
