# Supplementary material for: Clonal integration benefits Calystegia soldanella in heterogeneous habitats
Source: AoB Plants. 2024 May 20;16(3):plae028. doi: 10.1093/aobpla/plae028 (PMC11161862; doi:10.1093/aobpla/plae028)
Supplement: plae028_suppl_Supplementary_Table_S2 [file plae028_suppl_supplementary_table_s2.docx]

Table S2 Results of generalized linear mixed models for effects of environment, ramets states and their interaction on the growth index of whole clone of *Calystegia soldanella*

|  |  | SN | | SL(cm) | | ARN | | ARL(cm) | | URN | |
| --- | --- | --- | --- | --- | --- | --- | --- | --- | --- | --- | --- |
| Effcet | *df* | *F* | *P* | *F* | *P* | *F* | *P* | *F* | *P* | *F* | *P* |
| Fixed factor |  | | | | | | | | | | |
| E | 1 | 2.015 | 0.167 | 0.804 | 0.378 | 0.284 | 0.599 | 0.176 | 0.678 | 4.831 | **0.037** |
| R | 1 | 0.613 | 0.44 | 0.402 | 0.532 | 0.066 | 0.799 | 3.443 | 0.074 | 0.096 | 0.759 |
| E × R | 1 | 0.441 | 0.512 | 2.8 | 0.106 | 0.284 | 0.599 | 0.686 | 0.415 | 0.64 | 0.431 |
| Random factor |  | | | | | | | | | | |
| Block | 1 | 0.002 | 0.965 | 0.258 | 0.615 | 1.616 | 0.215 | 0.89 | 0.354 | 0.331 | 0.57 |

E: environment; R: ramets states; SN: spacers number; SL: spacers length; ARN: aboveground ramets number; ARL: aboveground ramets length; URN: underground ramets number
